# ØCD: protocol for the development and evaluation of a cognitive-behavioral prevention program for obsessive-compulsive disorder

**DOI:** 10.1186/s12888-024-05791-3

**Published:** 2024-05-09

**Authors:** Roxana A. I. Cardoș, Elisa P. Dumitru, Oana A. David

**Affiliations:** 1 The International Institute for the Advanced Studies of Psychotherapy and Applied Mental Health, Department of Clinical Psychology and Psychotherapy, Babeş-Bolyai University, Cluj-Napoca, Romania; 2 The International Institute for the Advanced Studies of Psychotherapy and Applied Mental Health, Doctoral School “Evidence-based assessment and psychological interventions”, Babeș-Bolyai University, No. 37, Republicii Street, 400015 Cluj-Napoca, Cluj, Romania

**Keywords:** Prevention, OCD, CBT

## Abstract

**Background:**

Obsessive-compulsive disorder (OCD) imposes significant burdens on individuals, families, and healthcare systems and the COVID-19 pandemic appears to have exacerbated OCD symptoms. Currently, there are no validated prevention programs for OCD, highlighting a critical gap in mental health services. This study aims to develop and validate the first ØCD prevention program, for at-risk adults, utilizing cognitive-behavioral therapy (CBT) and exposure response prevention (ERP) techniques.

**Methods:**

A single-blind, randomized controlled trial comparing the ØCD prevention program to a waitlist control group will be conducted. Participants, at-risk adults (18–65 years) with subclinical OCD symptoms (OCI-R score ≥ 12), will be recruited for the study. The ØCD prevention program compresise of six online group sessions incorporating CBT and ERP techniques over three modules. The primary outcomes are OCD symptom severity (measured by the Obsessive-Compulsive Inventory- revised form; OCI-R), depression symptoms (measured by the Patient Health Questionnaire; PHQ-9), and anxiety symptoms (measured by the Generalised Anxiety Disorder 7-item; GAD-7). Secondary outcomes include OCD-related beliefs, experiential avoidance, resilience, quality of life, uncertainty intolerance, automatic thoughts, and distress. Outcome measures will be collected at baseline, at completion of the intervention, and one year later (follow-up). At follow-up, we will also analyze the OCD diagnostic incidence, using the Structured Clinical Interview for DSM-5. We will employ a multivariate analysis of variance (MANOVA) to explore whether significant differences exist between groups across dependent variables. To compare the OCD incidence levels from the pre-test to the follow-up we will use the chi-squared test.

**Discusion:**

The present study may contribute novel data on the efficacy of OCD prevention approaches, leading to the development of an evidence-based OCD prevention program that could alleviate individual and societal burdens associated with OCD.

**Trial registration:**

This trial was approved by the University Ethical Review Authority (937/ 28.11.2023) at Babeș-Bolyai University and is registered on clinicaltrials.gov (ID: NCT06262464).

## Article summary

Strengths and limitations of this study

• First study to develop and validate an OCD prevention program for at-risk adults.

• The program integrates psychological prevention techniques from evidence-based treatments.

• Moderate expected rates of participants dropout

## Background

Obsessive-compulsive disorder (OCD) is one of the most debilitating mental health disorders, with considerable burden on patients, their families, and health services [19]. As a psychiatric disorder, it is characterized by the co-occurrence of recurring, intrusive obsessions that induce substantial distress, along with compulsions—ritualistic behaviors aimed at relieving the distress triggered by the obsessions but ultimately resulting in additional distress and impairment (American Psychiatric Association, 2013). The prevalence estimates vary from 0.7-3% in adults [26],Adam et al., 2011), and 0.25-0.30% in children (Sadler et al., 2017; [17].

OCD has both direct costs, such as medical costs [16, 18], as well as indirect costs, such as work absenteeism due to impaired functionality and lost opportunities for career advancement [9]. One UK study [19] found that the overall cost-of-illness of OCD rises to £5,095,759,464, with an average annual cost-of-illness of £7077 per person.

Worryingly, the COVID-19 pandemic seems to have led to an increase in the incidence of OCD. A systematic review [22] found that both people with and without diagnosed OCD before the pandemic experienced a worsening of OCD symptoms during the pandemic. One study [25] found that participants reported significantly higher OCD scores during the pandemic compared to before the pandemic, as OCD severity increased on all symptom dimensions and the most pronounced change was on the washing dimension. Another review (Cunning & Hodes, 2021) found that the COVID-19 pandemic is associated with a worsening of OCD symptoms in young people and that being in treatment seems to have a protective effect, thus underlining the importance of maintaining mental health services. While we have multiple evidence-based treatments for OCD, there are currently no validated protocols for the prevention of general OCD. This is a major shortcoming, seeing as an effective prevention program for at-risk individuals could constitute a preemptive strike against the high associated costs and burden of living that come with a diagnosis of OCD. Former prevention programs for depression and anxiety usually carried out for adolescents and young adults, have varying rates of efficacy, but are overall considered successful in reducing psychopathological symptoms incidence in time (Conley et al., 207; Werner et al., 2021).

The development and validation of an intervention protocol for the prevention of OCD among at-risk adults, as presented in the ØCD prevention program, are thus paramount. By advancing OCD preventive approaches, this study offers hope for mitigating the long-term consequences of OCD and reducing the associated costs and burden on individuals and society.

### Specific contents of the ØCD prevention protocol

The program involves six sessions and includes established components of CBT [4] and ERP [11], namely: (1) psychoeducation and encouragement to reduce, change, or eliminate anxiety-reducing rituals (1 session), (2) cognitive restructuring by challenging dysfunctional beliefs (2 sessions), (3) behavioral changes by prolonged, imaginal and real-life exposure to distress-provoking stimuli (2 sessions), (4) addressing ways and skills to reduce relevant risk factors (i.e. fear of COVID-19) and to develop resilience factors (i.e. resilience levels, coping skills) and summary and recapitulation (1 session). The six sessions of the 2 weeks program correspond to three treatment modules (3 hours/module). An overview of the content covered in each stage is provided in Table 1.

**Table 1:** Description of the ØCD modules and sessions

Module	Session	Session Content
Psychoeducation	Overview of OCD	• • Elicit from participant their understanding of OCD. • • Increase motivation for change through psychoeducation. • • Explain what OCD is and the forms it can take. • • Homework: monitor OCD-related thoughts, feelings, and behaviors.
1. Cognitive approach	2. Identifying obsessive sequences	• Mindfulness exercise (Body Scan) to ensure a relaxed state. • Homework debriefing. • Explain how obsessive sequences take place based on participants’ examples. • Connect obsessive sequences to their corresponding compulsions. Homework: identify obsessive sequences in everyday life.
2. Cognitive approach	3. Perspective and how to change it	• Homework debriefing. • Introduce the concept of perspective. • Explain how perspective can affect the formation and maintenance of the circle of obsessions. • Offer examples as to how changing the perspective can prevent engaging in the obsessive sequence and compulsions. • Introduce the Cognitive ABC model. • Homework: identify three cognitions and apply the Cognitive ABC model.
2. Behavioral approach	4. Exposure and Response Prevention (ERP)	• Homework debriefing. • Revision of the concepts discussed previously. • Discussion about compulsions. • Introduce Exposure and Response Prevention. • Engage participants in an ERP exercise. • Homework: choose a medium-level anxiety situation and apply the ERP steps.
3. Behavioral approach	5. Imaginal exposure and ERP	• Check in with the participants and their progress. • Recapitulate ERP and homework debriefing. • Introduce imaginal exposure. • Imaginal exposure exercise: participants will have 10 minutes to write a scenario about a situation that causes them anxiety. • Participants will monitor thoughts, anxiety levels, safety behaviors and strategies used to prevent engaging in the compulsions. • Homework: vocally record the scenario and listen to it 3-4 times after performing the Body Scan exercise.
3. Summary	6. Values and Committed Action. Summary	• Homework debriefing. • Revision of cognitive and behavioral skills acquired in the intervention. • Introduce Values and Committed Action. • Explain the importance of engaging in behaviors consistent with their values. • Write a values hierarchy and establish the behavioral changes needed to align behavior with values. • Create a maintenance plan for participants to maintain the progress made during the intervention. • Debriefing of the participant's experience in the intervention.

### Aim of the present study

The main objective of the proposed study is to develop and assess the efficacy of the ØCD prevention program in preventing OCD symptoms and reducing associated symptoms versus a waitlist control group. In this single-blind, superior randomized controlled trial, we will evaluate the efficacy of the ØCD prevention program compared with a waitlist. If, as we hypothesize, the ØCD prevention program demonstrates superiority compared to a control group, it has the potential to increase the variety of evidence-based intervention options for adults at risk of developing OCD.

We hypothesize that the ØCD prevention program will be superior to a control group in the reduction of OCD symptoms as measured by the Obsessional Compulsive Inventory – revised form (OCI-R; [10]), depressive symptoms (Patient Health Questionnaire-9,PHQ-9; [20]), and anxiety symptoms (Generalized Anxiety Disorder-7,GAD-7; [29]). The intervention also covers the cognitive component of OCD, so we expect a reduction in obsessive beliefs (The Obsessive Beliefs Questionnaire,OBQ-44; [24]) and automatic thoughts (Automatic Thoughts Questionnaire,ATQ; Steven & Phillip, 1980). We also expect a reduction in experiential avoidance levels (The Brief Experiential Avoidance Questionnaire; EAQ; Gamez et al., 2014), intolerance of uncertainty (Intolerance of Uncertainty Scale: IUS; [5]), and general distress (General Health Questionnaire-12,GHQ; [15]). As for protective factors, we expect an increase in resilience (Resilience Scale for Adults,RSA; [13]) and quality of life (WHO Quality of Life - BREF,WHOQOL-BREF; [28]).

## Methods

### Study design and ethical aspects

We will conduct a single-blind (blinded outcome assessors), randomized (1:1), controlled, parallel-group, superiority trial comparing the ØCD prevention program with a control group (waitlist) for adults at risk of OCD. The study is registered on clinicaltrials.gov (ID: NCT06262464). All quality and safety aspects will be regularly monitored by an external party: the University Ethical Review Board, and by the financing authority of the project (National Authority for Scientific Research). The study has been approved by the University Ethical Review Authority (937/28.11.2023). Potential protocol modifications will be described in detail on clinicaltrials.gov. We used the SPIRIT reporting guidelines [6].

Participants will be asked to provide informed consent before baseline assessment. They will receive detailed information about the study and their right to withdraw from the study without the obligation to give reasons. We do not expect major relevant risks for participants during the study, or after the study is completed. The time burden for the participants will be a reasonable amount. The data collection and storage will be conducted according to GDPR legislation, and all personal data will be securely stored on the University’s encrypted servers, following the set data management guidelines.

### Sample size

**Fig. 1:**
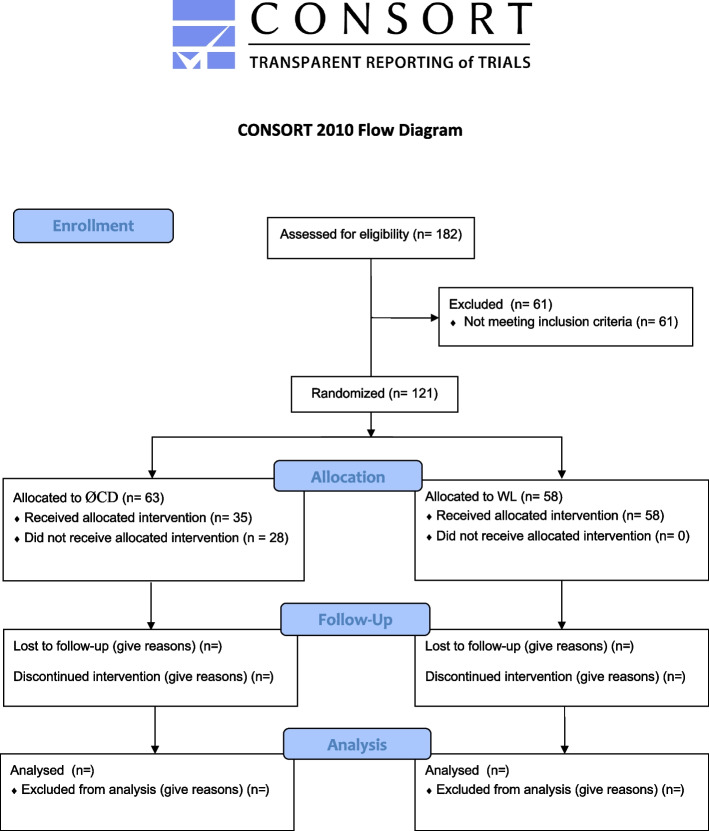
CONSORT flow diagram

G*Power was used for the sample size calculation and, following an effect size of d=0.3 with a n alpha level of α = 0.05 and power of 0.95, a sample size of 175 was suggested. Based on this power analysis and expected drop-out rates, we aim for 200 participants (100 per treatment arm). The Consolidated Standards of Reporting Trials flow chart of the trial is shown in Figure 1.

### Recruitment

**Fig. 2:**
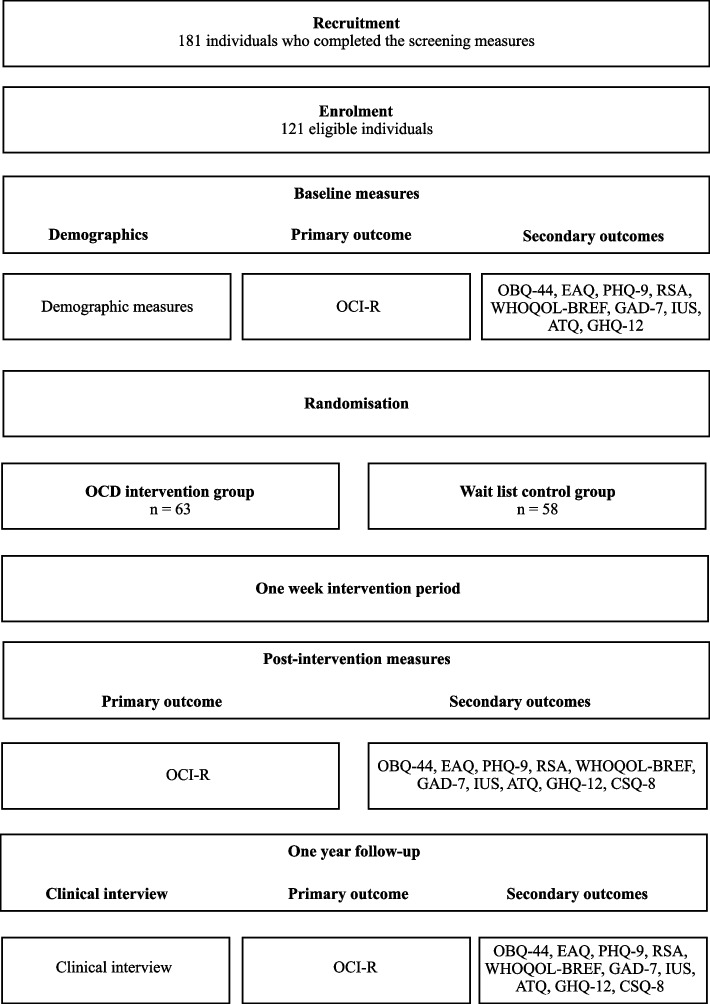
Planned flow of participants. Note: ATQ-Automatic Thoughts Questionnaire; BEAQ-Brief Experiential Avoidance Questionnaire; CSQ-8- Client Satisfaction Questionnaire-8; GAD-7-Generalized Anxiety Disorder-7; GHQ-12-General Health Questionnaire-12; IUS-Intolerance of Uncertainty Scale; OBQ-44-Obsessive Beliefs Questionnaire-44; ØCD - OCD prevention program; OCI-R-Obsessional Compulsive Inventory – Revised; PHQ-9-Patient Health Questionnaire-9; RSA-Resilience Scale for Adults; WHOQOL-BREF-WHO Quality of Life – BREF

Participants are recruited online, using social media platforms to promote the study. By clicking the enrolment link, participants read the study information section and the informed consent form, which can be signed digitally, by agreeing to the terms and the conditions of the study. Participants are queried about the criteria for inclusion and exclusion, following which eligible individuals are selected through randomization. The planned flow of participants can be consulted in Figure 2.

Therapists are from the clinical psychology master's program. We recruited four terminal year students following an introductory meeting on the purpose of the study and the procedures involved. The expected time expenditure for the therapists will be 9 hours per week. Before implementation, all therapists will have participated in a training supported by the study coordinator.

### Inclusion and exclusion criteria

Participants are included if they are of legal age (above 18). We include participants who have an OCI-R [10] score at or above the cut-off point on the OCD subscale (≥ 12). Exclusion criteria are as follows: (1) clinically diagnosed OCD, (2) current psychiatric/psychotherapeutic treatment, (3) a personality disorder diagnosis, and (4) suicidal ideation.

### Procedure and randomization

Inclusion and exclusion criteria are verified based on the screening form completed by the participants. Eligible participants are randomly allocated to one of the two conditions in a 1:1 ratio. Randomization is conducted by a member of the research team, using the online tool, randomizer.org. The allocation sequence is generated through a computer-generated random sequence for the two study conditions. The following study personnel will remain blind to group allocation until the final data analyses are completed: principal investigator, data managers, and outcome assessors.

### Outcome measures

For participants, the baseline assessment will take place after randomization and include a set of validated questionnaires.

### Primary outcome measures

#### Obsessional Compulsive Inventory - Revised (OCI-R; [10])

OCI-R is a self-report questionnaire of 18 items, and it measures OCD symptoms across six subscales: washing, checking, neutralising, obsessing, ordering, and hoarding. OCI-R was used in the initial screening of eligible participants, where the OCD subscale (which excludes the hoarding component) was used to determine participants who are at risk of developing OCD symptoms (cut off point (≥ 12). For the present study, the reduction in OCI-R OCD subscale assessment is the primary outcomes.

#### Patient Health Questionnaire-9 (PHQ-9; [20])

PHQ-9 is one of the most widely used measures of depression and it is based on the diagnostic criteria for major depressive disorder in the Diagnostic and Statistical Manual (DSM-IV; APA, 1994). Scores vary from 0 to 27, where a greater score indicates more severe depressive symptoms.

#### Generalized Anxiety Disorder-7 (GAD-7; [29])

GAD-7 measures symptoms of anxiety, based on the generalized anxiety disorder criteria in DSM-IV; APA, 1994). Respondents evaluate the level of their symptoms over the last two weeks and a cut-off score greater than 10 indicates further evaluation is recommended.

### Secondary outcome measures

#### Obsessive Beliefs Questionnaire (OBQ-44; [24])

OBQ-44 is a self-report scale that measures belief domains linked to OCD. The three subscales are (1) responsibility/threat estimation, (2) perfectionism/certainty, and (3) importance/control of thoughts. Answers are rated on a 7-point scale (1 = “disagree very much” to 7 = “agree very much”).

#### Automatic Thoughts Questionnaire (ATQ; Hollon & Kendall, 1980)

The Romanian version of ATQ [23] is a 15-item measure that evaluates self-reported negative thoughts. The questionnaire was developed to measure the most frequent negative thoughts and negative self-evaluations, typically associated with depression. Answers are rated on a 5-point scale (1 = “not at all” to 5 = “all the time”) and the total score ranges from 15 to 75.

#### Brief Experiential Avoidance Questionnaire (BEAQ; Gamez et al., 2014)

The BEAQ is a 15-item self-reports questionnaire that measures experiential avoidance. Items are rated on a 6-point Likert scale (1 = “strongly disagree” to 6 = “strongly agree”). Item 6 is a reversed item, and scores range from 15 to 90 points. Higher scores reflect higher experiential avoidance.

#### Intolerance of Uncertainty Scale (IUS; [5])

The IUS is a 27-item measure that evaluates how individuals relate to uncertainty. It contains items such as “Uncertainty makes me uneasy, anxious, or stressed”, “A small unforeseen event can spoil everything, even with the best of planning” and “When I am uncertain, I can’t go forward”. Answers are rated on a 5 points scale (1 = “not at all characteristics” to 5 = “entirely characteristic”)

#### General Health Questionnaire-12 (GHQ-12; [15])

The GHQ-12 consists of 12 items, each assessing the severity of a mental issue over the past few weeks. Answers are rated on a 4-point Likert scale, from 0 to 3. A total score of 36 can be generated, by rating the positive items from 0 (always) to 3 (never) and the negative ones from 3 (always) to 0 (never), so that a higher score indicate worse health.

#### Resilience Scale for Adults (RSA; [13])

The RSA is a 43-item measure with five factors: personal competence, social competence, family cohesion, social support, and personal organization. Answers are rated on a 5-point Likert scale from 1 = “not at all” to 5 = “a lot”).

#### WHO Quality of Life - BREF (WHOQOL-BREF; [28])

WHOQOL-BREF is one of the most widely used assessments of quality of life. In the current study, we will use the global item of the instrument (How would you rate your quality of life), answered on a 5-point Likert scale.

Post-intervention assessment will take place immediately after the intervention is completed and will include the same measures, as well as the Client Satisfaction Questionnaire-8 (CSQ-8; [21]). The assessments will be completed online, using the Qualtrics platform. We will also perform one follow-up assessment six months later, using the same measures and the Structured Clinical Interview for DSM-5 to compare the OCD diagnostic incidence.

### Statistical analyses

#### Data analysis

Demographic and clinical data will be reported as means and SDs for continuous parametric data, medians, and ranges for non-parametric data, and frequencies and percentages for categorical data. Only two authors will have access to the final trial dataset. To illustrate participant flow, we will report results in a CONSORT diagram. Data analysis will be conducted using SPSS for Windows version 25. Given the anticipated dropout rate, mixed models will be employed as the most suitable statistical method. This approach is well-suited for analyzing repeated measures, accounting for the dependency between observations and handling missing data effectively. Mixed models will be adjusted for baseline values of the repeated measures. Descriptive statistics will be generated to summarize the means and proportions of baseline clinical and demographic variables across treatment conditions, allowing for the assessment of potential imbalances. Fixed effects in the model will include time, treatment, and their interaction. In cases of missing data, these variables will also be included as fixed effects. The significance of the time*treatment interaction p-values for various outcome measures will be assessed using the Benjamini–Hochberg procedure (two-sided p < 0.05). Time will be treated categorically, with the first post-baseline measurement serving as the reference category. To address the correlation among repeated measurements, a first-order autoregressive (AR (1)) structure will be imposed on the residuals. Furthermore, the interaction effect between time and group will be explored by analyzing estimated marginal means at different time points. We anticipate a significant interaction effect between time and group, signifying differential changes in scores over time between the two treatment conditions. Specifically, we expect a more pronounced decline in scores over time in the prevention group compared to the control group. To assess the statistical significance of improvements in the treatment conditions, a least significant difference test will be conducted with estimated marginal means to compare changes between groups. Linear mixed models are expected to reveal a statistically significant decline in the prevention condition from pretest to post-test, with no significant differences between post-test and follow-up measures. To facilitate comparisons with other studies, Cohen’s d statistic will be calculated to determine within-group effect sizes. In cases where the proportion of missing data exceeds 10%, maximum likelihood imputations will be performed based on demographics and pretreatment scores. Additionally, the proportions of dropouts and diagnostic incidence (from pre-test to follow-up) in the two treatment groups will be compared using the chi-square test.

## Discussion

According to the most recent clinical advances guide for OCD [12], a greater effort needs to be made at multiple levels (e.g., education, treatment services development, and screening of ‘at-risk’ individuals) to implement effective strategies for prevention, early diagnosis, and intervention. To the best of our knowledge, we will develop the first evidence-based cognitive-behavioral prevention program for OCD for at-risk individuals. The study will provide novel data about the efficacy of OCD preventive strategies. Implementing the prevention program in normal healthcare would significantly enhance the availability of effective prevention programs to persons at risk of developing OCD. The potential benefits of such a prevention program extend beyond individual participants to encompass broader societal and research impacts. By intervening early to prevent the onset of OCD symptoms, the program could alleviate strain on mental health services, reduce healthcare costs, and improve overall well-being. Moreover, the research generated from implementing and evaluating this innovative prevention approach could inform future interventions and contribute to advancing our understanding of OCD prevention.

### Trial status

Inclusion started in March 2024 and is expected to end in October 2024. At the time of submission, 121 participants had been randomized. Data analysis and reporting of results will begin when all data from the primary endpoint has been collected.

## Data Availability

Data will be made available upon reasonable request from the corresponding author.

## Abbreviations

OCD: Obsessive Compulsive Disorder
CBT: Cognitive Behavioral Therapy
ERP: Exposure and Response Prevention
